# Oxyanion induced variations in domain structure for amorphous cobalt oxide oxygen evolving catalysts, resolved by X-ray pair distribution function analysis

**DOI:** 10.1107/S2052520615022180

**Published:** 2015-12-01

**Authors:** Gihan Kwon, Oleksandr Kokhan, Ali Han, Karena W. Chapman, Peter J. Chupas, Pingwu Du, David M. Tiede

**Affiliations:** a Chemical Sciences and Engineering Division, Argonne National Laboratory, 9700 South Cass Ave, Lemont, IL 60439, USA; b Department of Materials Science and Engineering, University of Science and Technology of China, 96 Jinzhai Rd, Hefei 230026, People’s Republic of China; c X-ray Science Division, Argonne National Laboratory, 9700 South Cass Ave, Lemont, IL 60439, United States

**Keywords:** water-splitting catalysts, amorphous metal oxides, pair distribution function analysis, high-energy X-ray scattering, cobalt oxide

## Abstract

Amorphous thin film oxygen evolving catalysts, OECs, of first-row transition metals show promise to serve as self-assembling photoanode materials in solar-driven, photoelectrochemical ‘artificial leaf’ devices. This report demonstrates the ability to use high-energy X-ray scattering and atomic pair distribution function analysis, PDF, to resolve structure in amorphous metal oxide catalyst films. The analysis is applied here to resolve domain structure differences induced by oxyanion substitution during the electrochemical assembly of amorphous cobalt oxide catalyst films, Co-OEC. PDF patterns for Co-OEC films formed using phosphate, Pi, methylphosphate, MPi, and borate, Bi, electrolyte buffers show that the resulting domains vary in size following the sequence Pi < MPi < Bi. The increases in domain size for CoMPi and CoBi were found to be correlated with increases in the contributions from bilayer and trilayer stacked domains having structures intermediate between those of the LiCoOO and CoO(OH) mineral forms. The lattice structures and offset stacking of adjacent layers in the partially stacked CoMPi and CoBi domains were best matched to those in the LiCoOO layered structure. The results demonstrate the ability of PDF analysis to elucidate features of domain size, structure, defect content and mesoscale organization for amorphous metal oxide catalysts that are not readily accessed by other X-ray techniques. PDF structure analysis is shown to provide a way to characterize domain structures in different forms of amorphous oxide catalysts, and hence provide an opportunity to investigate correlations between domain structure and catalytic activity.

## Abstract 2

Amorphous thin-film oxygen evolving catalysts, OECs, of first-row transition metals show promise to serve as self-assembling materials in solar-driven, photoelectrochemical ‘artificial leaf’ devices. This report demonstrates the ability to use high-energy X-ray scattering and atomic pair distribution function analyses, PDF, to resolve structure in amorphous metal oxide catalysts films, and is applied here to resolve domain structure differences induced by oxyanion substitution during the electrochemical assembly of amorphous cobalt oxide catalyst films.

## 1. Introduction   

Solar hydrogen production from water has been recognized as an attractive process to produce carbon-neutral, renewable fuel, but its development requires cheap and efficient water oxidation catalysts. In nature, water oxidation is catalysed by a CaMn_4_O_*x*_ active site in photosystem II that is continuously regenerated using photo-oxidative chemistry during prolonged catalytic cycling (Dasgupta *et al.*, 2008 ▸; Nixon *et al.*, 2010 ▸; Becker *et al.*, 2011 ▸). Electrodeposited amorphous oxide films of cobalt (Kanan *et al.*, 2009 ▸; Kanan & Nocera, 2008 ▸), nickel (Dincă *et al.*, 2010 ▸), manganese (Iyer *et al.*, 2012 ▸; Baktash *et al.*, 2013 ▸; Najafpour *et al.*, 2013 ▸), iridium (Blakemore *et al.*, 2011 ▸) and mixed metals (Smith, Prévot, Fagan, Trudel & Berlinguette, 2013 ▸; Smith, Prévot, Fagan, Zhang *et al.*, 2013 ▸; Burke *et al.*, 2015 ▸; Trotochaud *et al.*, 2014 ▸) have attracted much attention because of the simplicity of electrochemical deposition of catalytic thin-films from metal salt solutions. In particular, cobalt-based amorphous oxide catalytic films formed in the presence of inorganic phosphate, CoPi, show remarkable robustness that is in part due to the biomimetic oxidative regeneration of the catalyst (Kanan & Nocera, 2008 ▸; Kanan *et al.*, 2009 ▸; Lutterman *et al.*, 2009 ▸; Ullman & Nocera, 2013 ▸). Resolution of the chemistries underlying Co-OEC amorphous film assembly and mechanisms for catalysis are important for developing solar fuels technologies based on artificial photosynthesis, and have possible additional relevance to the assembly and function of the CaMn_4_O_*x*_ water-splitting catalyst cofactor in photosynthesis (Pace *et al.*, 2012 ▸; Nocera, 2012 ▸; Symes *et al.*, 2011 ▸; Swiegers *et al.*, 2011 ▸; Esswein *et al.*, 2011 ▸; Dau *et al.*, 2010 ▸; Mattioli *et al.*, 2013 ▸).

The structure and catalytic function of electrochemically deposited cobaltate films have been found to depend critically upon pH and the chemical character of the electrolyte (Gerken *et al.*, 2011 ▸). Oxidation of cobalt salts in the presence of proton-accepting oxyanion electrolytes, and particularly with phosphate (Pi), methylphosphate (MPi) and borate (Bi) were shown to yield amorphous cobaltate films with enhanced water-splitting catalytic function (Kanan & Nocera, 2008 ▸; Surendranath *et al.*, 2009 ▸; Esswein *et al.*, 2011 ▸). Key questions remain to understand how structures in amorphous metal oxides are altered and linked to improved catalytic function, and to resolve the sites and mechanisms for water-splitting catalysis.

Combined electrochemistry and spectroscopic analyses of amorphous Co-oxide films have proposed that water oxidation follows from charge accumulation in the domains which progresses to the point where Co^IV^–(di-μ-oxo)–Co^IV^ redox pairs are formed with Co atoms having terminally coordinate oxygen ligands, and that these are the active sites for water-splitting catalysis (Surendranath *et al.*, 2010 ▸; Risch *et al.*, 2015 ▸). Line-shape changes in XAFS data have shown that cobaltate lattice domains formed with acetate or chloride electrolytes have increased order compared with those formed in the presence of phosphate, and that this increased order is correlated with decreases in catalytic current densities (Risch *et al.*, 2012 ▸). These findings have been noted to provide circumstantial support for the hypothesis that catalysis is linked to terminal oxygen ligands at the domain edges (Risch *et al.*, 2012 ▸).

**Figure 1:**
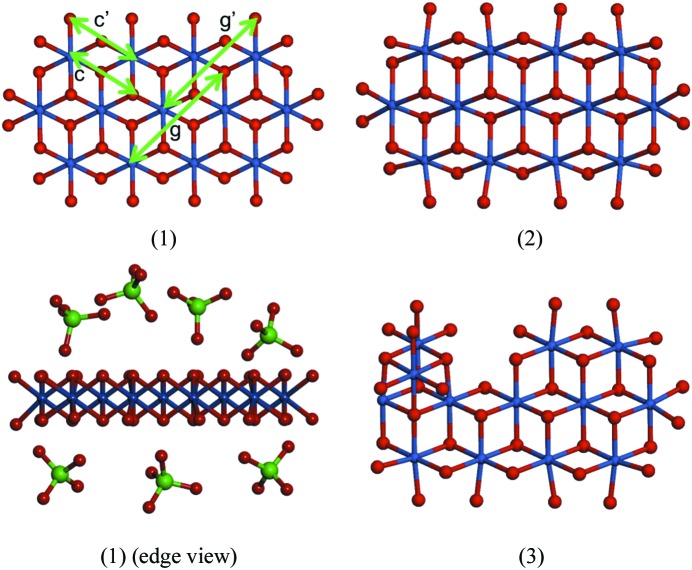
Cobaltate lattice models used to fit the PDF data for the CoPi (Du *et al.*, 2012 ▸). Structure (1) is a 13 Co atom containing lattice fragment from the LiCoOO structure. The labelled arrows (*c*, *g*) indicate selected Co—O atom pair distances involving terminal O atoms (*c*′, *g*′) that required refinement to fit experimental PDF data. Structures (2) and (3) are examples of refined models having distortions in the coordination geometry for the terminal O atom and including defect sites, respectively, that provided improved fits to PDF data (Du *et al.*, 2012 ▸). Structure (1) is also shown in an edge-on view, illustrating the surface location of Pi found to be disordered by PDF (Du *et al.*, 2012 ▸), and resolved by NMR measurements to be disordered, but located above and below the lattice surface plane (Harley *et al.*, 2012 ▸).

High-energy X-ray scattering with atomic pair distribution function (PDF) analyses are a useful complement to XAFS analysis, and provide a means to more probe domain size, structure and extent of mesoscale ordering. PDF measurements provide a measure of atom pair distances ranging from bonded atom pairs to nanoparticle dimensions (Juhás *et al.*, 2006 ▸; Billinge & Kanatzidis, 2004 ▸; Egami & Billinge, 2003 ▸; Chupas *et al.*, 2009 ▸; Malavasi, 2011 ▸; Mulfort *et al.*, 2013 ▸). PDF analysis from X-ray scattering measured with high signal-to-noise (> 10) to a scattering vector, *q*, up to 24 Å^−1^ on CoPi films formed rapidly during water oxidation (*E* = 1.34 V, pH 7, referenced to the normal hydrogen electrode, NHE) were found to approximately fit with a single structure composed of 13 Co atoms in a cobaltate lattice, (1) in Fig. 1 ▸ (Du *et al.*, 2012 ▸). Significantly these PDF measurements recorded with high spatial resolution, *d* = 0.29 Å, identified line shapes in the PDF pattern that could be fit using models having distortions in the coordination geometries at the domain edge, (2), or lattice defect sites, (3), and detected the presence of disordered Pi in the films (Du *et al.*, 2012 ▸).

Subsequent PDF measurements on CoPi formed following 14–16 h of deposition at lower potentials before the onset of catalysis, using *E* = 1.04 V (*versus* NHE), pH 7, found larger average CoPi domains corresponding to about 19 Co atoms (Farrow *et al.*, 2013 ▸). This work also characterized the catalyst film formed in the presence of borate, CoBi, which showed that the change in oxyanion was accompanied by an increase in domain size for the CoBi compared with the CoPi films, which was correlated with the appearance of a disordered cobalt oxyhydroxide layered domain structure in the CoBi (Farrow *et al.*, 2013 ▸). Particularly for thicker films, electrochemical measurements found that CoBi supported higher catalytic currents than CoPi (Farrow *et al.*, 2013 ▸). This finding showed that the catalytic activities of the CoPi and CoBi films are not directly correlated to the proportion of Co atoms in domain edges that are presumed to be the sites of catalysis, but rather may be correlated to differences in domain conductivities and charge accumulation properties in the two cobaltate films (Farrow *et al.*, 2013 ▸).

Herein we report on domain size and layering characteristics of Co-OEC films formed from a series of oxyanions: the potassium salts of Pi, MPi and Bi, and measured with sufficient spatial resolution to resolve contributions from edge or defect sites. The films were found to be composed of analogous cobaltate domains that increase in size, following the sequence of Pi < MPi < Bi with domains having maximum average atom-pair distances of approximately 13, 16 and 20 Å, respectively. The increases in domain size for CoMPi and CoBi were found to be correlated to a transition to mixtures that included layered domains with layer spacing and structure comparable to LiCoOO mineral forms. These layered domains could be distinguished from the structures of CoOOH, NaCoOO and KCoOO layered mineral domains and were shown to have limited stacking coherence length corresponding to mixtures of 1–3 layers. In contrast, the CoPi domains were constantly found to be not stacked, but instead correspond to small hydrous layers, consistent with a model for phosphate intercalation and possible relatively long-range disordered stacking (Harley *et al.*, 2012 ▸). Finally, coincident with increases in domain size, contributions from Co atoms with distorted coordination geometries modelled to be associated with domain edges and defect sites were found to diminish. These results show that PDF measurements provide quantitative markers for tracking domain size and structure, defect sites and mesoscale organization in different forms of amorphous oxide catalysts. This work suggests opportunities to use PDF as a means to investigate correlations between domain structure and catalytic function.

## 2. Experimental and methods   

Co-OEC films on the surface of ITO (indium tin oxide) were prepared by anodic electrochemical deposition from aqueous buffered solutions containing the oxyanions Pi, MPi or Bi, following the conditions described previously (Kanan & Nocera, 2008 ▸; Surendranath *et al.*, 2009 ▸). This involved electrochemical deposition by applying 1.34 V potential for 0.1 *M* potassium phosphate buffer, pH 7.0; by applying 1.28 V for 0.1 *M* potassium methylphosphate buffer, pH 8.0; and by applying 1.21 V for 0.1 *M* potassium borate buffer, pH 9.2. All potentials were referenced to the NHE, and the electrolyte solutions additionally contained 0.5 m*M* Co(NO_3_)_2_·6H_2_O. The electrolyses were stopped after 2–5 h deposition. The black deposited films were rinsed with water, dried, scrapped off the electrode surface and loaded as powders into 1 mm diameter thin-walled (10 µm) glass or Kapton capillaries. High-energy X-ray (58.66 keV, λ = 0.2114 Å) scattering patterns for the Co-oxide catalyst powders, capillaries and backgrounds were measured as a function of the scattered wavevector **q**, where *q* = 4π sin(θ)/λ and 2θ is the scattered angle, in the range 0.4 < *q* < 24 Å^−1^ at the Advanced Photon Source (APS) of Argonne National Laboratory at beamline 11-ID-B (Chupas *et al.*, 2003 ▸, 2007 ▸).

Experimental HEXS patterns were used to generate the pair distribution function *G*(*r*) using the program *PDFgetX2* (Qiu *et al.*, 2004 ▸). In this procedure, the experimental scattering patterns were corrected for solvent, container and instrument background scatterings, X-ray polarization, sample absorption and Compton scattering to yield the total scattering for the solute, *I*(*q*). The reduced scattering structure function *F*(*q*) was calculated from *I*(*q*) according to
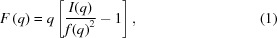
where *f*(*q*) is the sum of the composite atomic form factors, 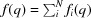. The real space pair distribution function, *G*(*r*), was obtained by direct Fourier transform of the oscillatory *F*(*q*)
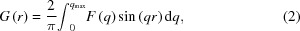
with *F*(*q*) extrapolated to *F*(0) below the experimental *q* range < 0.4 Å^−1^ and using *q*_max_ = 24 Å^−1^ and a Lorch dampening function to remove truncation effects (Qiu *et al.*, 2004 ▸). *G*(*r*) is related to the real space electron density distribution function according to
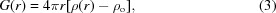
where ρ(*r*) is the spherical average of the real space electron density distribution function, ρ(*r*) and ρ_o_ is the average electron density of the sample (Chupas *et al.*, 2009 ▸; Juhás *et al.*, 2006 ▸; Qiu *et al.*, 2004 ▸; Billinge & Kanatzidis, 2004 ▸). The intensity of the high-energy APS beamline (Chupas *et al.*, 2003 ▸, 2007 ▸) allows *S*(*q*) to be measured with a signal-to-noise of greater than 10 for the Co-OEC powders throughout the measured *q* range, 0.4 < *q* < 24 Å^−1^. The level of spatial resolution was found to be necessary to resolve contributions from edge or defect sites in the CoPi films.

Models for Co-oxide domains in the water-oxidation catalyst films were built by extracting coordinates of variable dimensions from reference crystal structures, including layered LiCoO_2_, ICSD entry 172909 (Takahashi *et al.*, 2007 ▸), and CoOOH, ICSD entry 22285 (Delaplane, 1969 ▸). Scattering patterns, *S*(*q*) for the model domains were calculated from the atomic scattering parameters using the program *solX* (Tiede *et al.*, 2009 ▸; O’Donnell *et al.*, 2007 ▸; Zuo *et al.*, 2006 ▸), and the corresponding *G*(*r*) patterns were calculated from equation (1) as described previously (Mulfort *et al.*, 2013 ▸; Blakemore *et al.*, 2013 ▸; Du *et al.*, 2012 ▸).

SEM image and energy-dispersive X-ray analysis (EDX) data were collected with scanning electron microscopy (SEM, Hitachi S-4700-II with EDX detector, secondary electron detector and a backscatter electron detector) at the Electron Microscopy Center, Argonne National Laboratory. EDX data were acquired at 12 kV.

## 3. Results and discussion   

**Figure 2:**
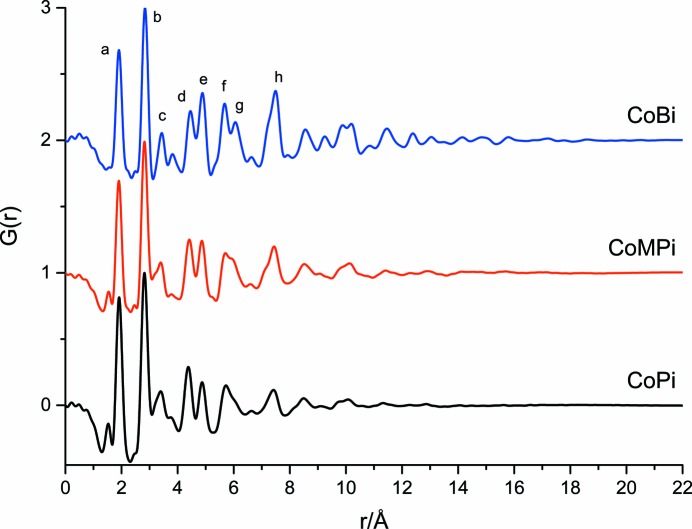
Pair distribution function, *G*(*r*), measured for the cobalt oxide water oxidation catalyst’s *ex situ* films grown from three different buffer solutions. The *G*(*r*) traces are arbitrarily vertically offset to increase visibility: bottom, Pi; middle, MPi; top, Bi. Individual peaks are labelled in the CoBi pattern, and are used to highlight specific atom pair correlation peaks discussed previously (Du *et al.*, 2012 ▸) and in the text.

Fig. 2 ▸ shows PDF patterns, measured as *G*(*r*) functions for Co-OEC formed in the presence of phosphate (pH = 7.0), methylphosphate (pH = 8.0) or borate (pH = 9.2) as the proton accepting oxyanion, following procedures described previously (Kanan & Nocera, 2008 ▸; Surendranath *et al.*, 2009 ▸; Esswein *et al.*, 2011 ▸). The corresponding experimental *S*(*q*) are shown in Fig. S1 of the supporting information. The morphologies and elemental compositions for the CoPi, CoMPi and CoBi OECs analysed in this study correlate to those reported earlier (Surendranath *et al.*, 2009 ▸; Kanan *et al.*, 2009 ▸; Kanan & Nocera, 2008 ▸). Representative scanning electron microscopy (SEM) images and EDX spectra with elemental composition ratio are shown in Figs. S2–S4.

For each Co-OEC, the first major peaks in the *G*(*r*) occur at 1.91 (2) and 2.81 (2) Å, with uncertainty from PDF measurements on four different CoPi samples electrochemically deposited and processed using equivalent conditions. These values correspond closely to the first coordination shell Co—O, 1.89 (1) Å, and μ_2_-oxo-bridged Co—Co, 2.81 (1) Å, distances, measured by XAFS (Mattioli *et al.*, 2011 ▸; Kanan *et al.*, 2010 ▸; Risch *et al.*, 2009 ▸). We note that the experimental *G*(*r*) pattern for the CoPi measured here reproduces the PDF features described previously (Du *et al.*, 2012 ▸), particularly with respect to the loss in intensities for peaks labelled *c* and *g* in Fig. 2 ▸ compared with the corresponding peak intensities calculated from cobaltate lattice structures. This can be modelled to arise from distortions in the coordination geometry for terminal O atoms, **2**, or lattice defect sites, **3**, as previously discussed (Du *et al.*, 2012 ▸). In addition, the *G*(*r*) pattern for the CoPi in Fig. 2 ▸ exhibits a peak at 1.5 Å that was shown to match the phosphate P—O distance with an amplitude corresponding to 7 phosphate groups per 13 Co atom domain (Du *et al.*, 2012 ▸). The *G*(*r*) intensity pattern for the P—O and Co—O bonded pairs is consistent with Co:P elemental analyses that report Co:P ratios in the range 2:1 to 3:1 (Surendranath *et al.*, 2009 ▸; Kanan *et al.*, 2009 ▸; Kanan & Nocera, 2008 ▸). The absence of a resolvable contribution of P–Co pairs at longer distances suggests that phosphate must be included as a disordered component in the CoPi-OEC (Du *et al.*, 2012 ▸).

Compared with the CoPi, *G*(*r*) measurements for the CoMPi and CoBi in Fig. 2 ▸ show a trend towards progressively longer maximum atom pair distances. Figs. S5 shows representative PDF patterns measured for pairs of different CoPi, CoMPi and CoBi films formed from equivalent electrolysis conditions. Some variability is seen in relative peak intensities. This variability could arise from a variety of causes, including inconsistencies in experimental background and other data corrections, variation in domain structure induced by the rinsing and dying procedures used to make the *ex situ* powders for PDF measurements, and presumably also from the heterogeneous nature of the amorphous films which may yield slightly varying distributions of domains in individual experiments. In spite of these variances, the PDF patterns show a clear trend for increasing the dimension of the domains following the sequence: Pi < MPi < Bi. This indicates an important role for the anions, or possibly the accompanying pH and electrochemical conditions, in determining the average domain size during film deposition. The longest atom-pair distances in the domain model (2) used to fit the PDF pattern (Du *et al.*, 2012 ▸) arise from Co—O and O—O atom pairs at 12.7 and 14.2 Å, respectively. This corresponds to the longest pair correlation seen for the CoPi to occur approximately at 13 Å. The longest resolved atom pair peak in the CoMPi is seen at about 16 Å, while for the CoBi the experimentally resolved peaks extend to approximately 20 Å, Fig. 2 ▸ and Figs. S5. The oxyanion dependent changes in Co-OEC domain size reported here, and those measured earlier for CoPi and CoBi (Farrow *et al.*, 2013 ▸), are in keeping with XAFS measurements that compared structures of Co-OEC when Pi is replaced by non-oxyanions, either acetate or chloride salts, during electrolysis (Risch *et al.*, 2012 ▸). In the case of the XAFS measurements, Pi substitution was shown to be correlated with a transition to a more ordered lattice, interpreted to be a result of an increased domain size, although domain dimensions cannot be directly accessed by XAFS data. The PDF results presented here provide a direct measure of the average change in Co-OEC domain size caused by the replacement of Pi with other oxyanions.

The PDF results obtained from HEXS data measured with a reciprocal space resolution of 24 Å^−1^ shown in Fig. 2 ▸ are also seen to allow detection of pair correlation peaks corresponding to the oxyanions. As discussed above, the P—O bond distance at 1.52 Å is resolved for CoPi, and with lower amplitude in the CoMPi. This amplitude difference is consistent with the lower oxyanion content in CoMPi and CoBi films (Surendranath *et al.*, 2009 ▸), and the diminished weighting of low *Z* B atoms for the CoBi PDF. We note that some variability in the detection of the oxyanion peaks, as illustrated by a comparison of representative difference sample preparations in Fig. S5. Pi, MPi and Bi oxyanions used as the electrolyte during film deposition might be variably retained in the *ex situ* powders used for PDF measurement. For example, Figs. S6 compares the PDF patterns for CoPi and CoMPi before and following overnight soaking in Milli-Q purified water.

In addition to changes in the domain size, the PDF patterns show characteristic progressive changes following the sequence CoPi, CoMPi to CoBi. One key feature of the CoBi pattern is that it demonstrates a sharper, more resolved feature than the CoPi pattern. For example, this is seen for peaks labelled *c* and *g* in Fig. 2 ▸. Diminished intensity for peaks *c* and *g* in the experimental CoPi PDF can be accounted for by introducing distortions in the coordination geometries for the terminal O atoms (Du *et al.*, 2012 ▸). The PDF data in Fig. 2 ▸ show that there is a progressive recovery of peaks *c* and *g* following the sequence CoPi, CoMPi to CoBi. For example, the amplitude of peak *c* is increased and an added feature is seen in CoBi compared with CoPi. Similarly, peak *g* is resolved separately in CoBi, but is an unresolved shoulder in CoPi. This is consistent with the assignment for the intensities of these peaks serving as markers for distorted coordination geometries for terminal and/or defect atom sites, which in turn make proportionally smaller contributions with the increase in domain size following the sequence CoPi, CoMPi to CoBi.

**Figure 3:**
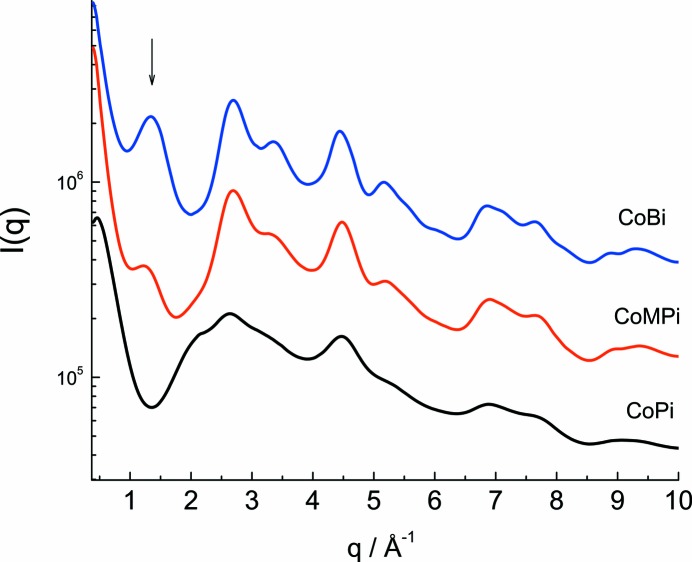
Experimental scattering patterns, *I*(*q*), measured for the cobalt oxide water oxidation catalyst *ex situ* films, CoPi, CoMPi and CoB, grown from three different buffer solutions. The *I*(*q*) traces are arbitrarily vertically offset to increase visibility: Pi, bottom; MPi, middle; Bi, top.

Fig. 3 ▸ shows unprocessed, azimuthally averaged detector scattering data, *I*(*q*), used for the PDF analysis and measured for CoPi, CoMPi and CoBi OECs. The identification of markers for structure changes in unprocessed experimental *I*(*q*) patterns is a useful check on structure characterization as it provides a means to recognize elements of structure change without the data processing and parameterization needed for PDF analysis. The experimental *I*(*q*) measured for each Co-OEC shows characteristic features, including a scattering peak at about *q* = 1.36 Å^−1^ which appears with increasing amplitude in the CoMPi and CoBi OEC, but is absent for the scattering data for the CoPi.

**Figure 4:**
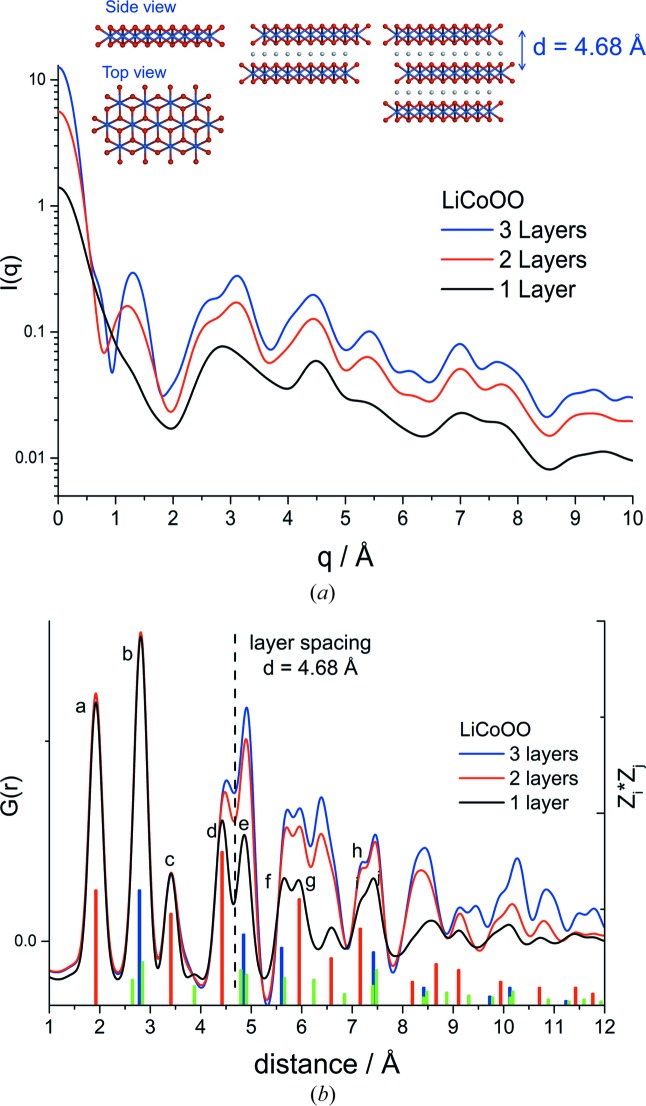
Calculated total scattering, *I*(*q*), (*a*), and PDF, *G*(*r*), (*b*), for a 13 Co atom domain, model **1**, as an isolated layer (black line), bilayer (red line), trilayer (blue line). The domain and stacking structures were extracted from the LiCoOO crystal structures. The inset in (*a*) shows the monolayer, bilayer and trilayer structures. The interlayer lithium ions were excluded from the calculation. (*b*) also includes a normalized, color-coded plot of the atom pair distances weighted by the product of the atomic numbers, *Z*_*i*_ · *Z*_*j*_, calculated for the **1** layer structure, with the colored bars red, blue and green bars corresponding to the sum of the Co—O, Co—Co and O—O atom pairs at each distance, respectively. The dashed line in (*b*) marks the layer spacing for the LiCoOO mineral structure.

The characteristic changes in the experimental *I*(*q*) and PDF patterns between CoPi, CoMPi and CoBi can be interpreted by comparison to *I*(*q*) and PDF calculated for domain structures extracted from layered cobaltate mineral structures. For example, Fig. 4 ▸ illustrates characteristic features introduced by including domain stacking on *I*(*q*) and *G*(*r*) calculated using the LiCoOO crystal structure (Takahashi *et al.*, 2007 ▸). In this example, *I*(*q*) and *G*(*r*) were calculated for domain model **1** in monolayer, bilayer and trilayer stacked assemblies, keeping the domain size constant in each layer, and using layer positioning determined by the LiCoOO crystal structure. The domain model structures are illustrated in the inset in Fig. 4 ▸(*a*), top. The interlayer lithium ions were excluded from the calculations. The calculated *I*(*q*) patterns, Fig. 4 ▸(*a*), show that the introduction of domain stacking is correlated with the appearance of a new scattering peak at a small angle, *q* = 1.31 Å^−1^. The line shape and position of this peak is a function of the number of layers in the stack and the inter-layer distance. By extending the assembly beyond three layers, the interlayer interference is reinforced and the first scattering interference peak narrows and progressively evolves into a Bragg diffraction layer peak. The shape of the first scattering peak can be altered and additional scattering features can be introduced by adding slip-stack offsets along the crystallographic **a** and **b** directions or by varying the dimensions of individual layers in the bilayer and trilayer stacks. However, the symmetry of the experimental *I*(*q*) first interference peak for CoBi and CoMPi suggests that these effects are largely averaged out, and that the first interference peak in the *I*(*q*) provides an experimental marker for layering in amorphous cobalt oxides.

The sensitivity of the scattering peaks as markers for domain stacking is shown in Figs. S7 by a comparison of experimental scattering for the CoPi, CoMPi and CoBi OEC to calculated scattering curves using the reference model **1** monolayer, bilayer, trilayer domains, but applied to CoOOH and LiCoOO mineral structures. The stacking of CoOOH and LiCoOO differ by the inter-layer distance, 4.38 and 4.68 Å, respectively, and have different offset alignment between adjacent layers. For both models the introduction of domain stacking is associated with the appearance of an additional scattering peak in the low *q* region, with the peak position correlated to the inter-layer spacing. Model domains composed of four and more layers gave linewidths and intensities which are narrower and more intense than those seen in the CoMPi and CoBi data. Variations are seen in the amplitude of the layering peak in CoBi in different samples, indicating that the extent of domain layering in the CoBi OEC is variable in different sample preparations. The correlation between this layering variation and catalytic activity is significant, and will be described in a subsequent publication. Here we focus primarily on developing the protocol for mesoscopic scale structure analysis.

For the data shown in Fig. 3 ▸, the CoBi sample can be roughly estimated to be composed of a mixture of monolayer, bilayer and trilayer domains with about 50% in the stacked form, based on the relative amplitude of the inter-layer scattering interference peak at *q* = 1.36 Å^−1^ compared with the interference peak at *q* = 2.7 Å^−1^. The amplitude and shifted peak position of the layered interference peak for the CoMPi compared with CoBi OEC can be interpreted in this context as a lower extent of stacking and a shift toward the smaller bilayer form in CoMPi. The absence of this peak for the CoPi confirms the monolayer character of this domain. We note here that the first *I*(*q*) interference peak for CoBi, *q* = 1.36 Å^−1^, is intermediate, but somewhat better aligned to CoOOH, *q* = 1.38 Å^−1^, than LiCoOO, *q* = 1.31 Å^−1^, tri-layer stacks. *I*(*q*) calculated from comparable models using the NaCoOO and KCoOO minerals have much larger interlayer spacing of 5.50 and 6.91 Å, respectively, and could be ruled out as candidate models for CoMPi and CoBi. In addition, the *I*(*q*) comparison shown in Fig. S7 suggests that the CoOOH stacked model is somewhat better aligned to the CoBi experiment in the low *q* region 2.0 < *q* < 4.5 Å^−1^. However, at higher *q* the *I*(*q*) for LiCoOO appears to be qualitatively closer to CoBi. For example, the CoOOH models have a *I*(*q*) peak at 9.5 Å^−1^, which is absent in the LiCoOO model and in the CoBi data. Both higher angle scattering features and effects of layered lattice structures can be further analysed in the PDF.

Fig. 4 ▸(*b*) shows the progressive change in calculated PDF patterns for LiCoOO domain models with different extents of stacking. This sequence shows analogies to those seen in the PDF for the experimental sequence CoPi, CoMPi and CoBi OEC, Fig. 2 ▸. In particular, a marker for stacking is seen by the increase in the amplitude of the peak labelled *e* compared with *d*, Fig. 4 ▸(*b*). This relative intensity change is also seen in the experimental PDF, Fig. 2 ▸. In this stacking model, the marker for edge atom distortions, identified by the relative amplitudes of peaks *g* compared with *f*, is still detected in the PDF for the layered structures. This is further shown by the analogous comparison of monolayer, bilayer and trilayer LiCoOO stacking models, but using individual layers having the edge distortions shown as in model **2**, Figs. S8. This confirms the presence of stacking within a LiCoOO model; layer stacking peaks do not interfere with the use of the PDF peak *g* as a marker for edge or terminal oxygen atom distortions. We note that the introduction of stacking in the LiCoOO model calculations is also correlated with several longer-range, inter-layer atom pair correlations. This pattern of additional pair correlations is not seen in the data. However, these longer-range pair correlations are dependent upon the details of the overlap, alignment and disorder parameters between adjacent layers. We expect that a quantitative modelling would need to include the effects of stacking alignment disorders.

The qualitative agreement between the sequence of PDF patterns for CoPi, CoMPi and CoBi OEC and stacking in the LiCoOO model can be contrasted with the markers for stacking in the CoOOH domain model. Fig. S9 shows the PDF patterns for the CoOOH domain model in monolayer, bilayer and trilayer stacks. Inter-layer pair correlation peaks are seen that overlap those from the lattice in the individual layers. The prominent distinction in PDF markers for stacking in CoOOH and LiCoOO domain models arises because of the differences in inter-layer *a*–*b* plane alignments in the mineral structures. These result in the shift in positions for the inter-layer atom pair distance peaks. Interestingly, these different PDF markers for domain stacking can be used to distinguish between CoOOH and LiCoOO domain stacking models. The comparison of experimental and calculated PDF patterns show that domains in the CoPi, CoMPi and CoBi OEC differ in domain size and extent of layering, and can be qualitatively described using a LiCoOO layered model. However, a quantitative model may need to consider structures with intermediate protonation states between CoOOH and LiCoOO.

**Figure 5:**
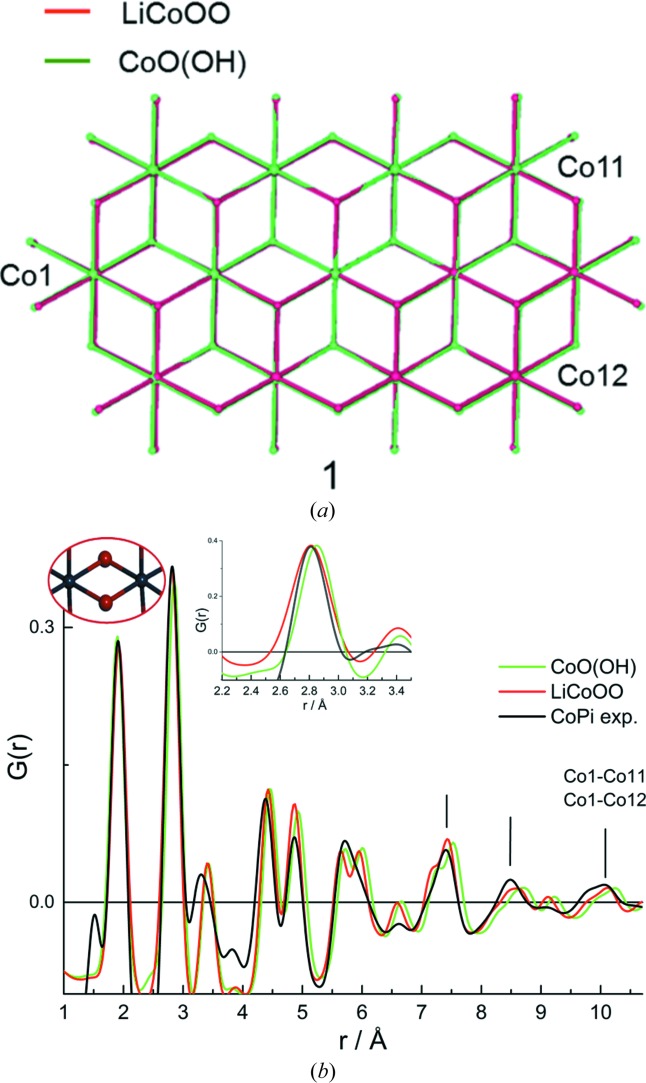
Comparison of LiCoOO and CoO(OH) lattice structures. (*a*) shows an overlap of the 13 Co atom domain model **1**, using the LiCoOO (red) and CoO(OH) (green) mineral structures, centered on the central Co atom. The overlap shows the slight expansion of the domain size for the CoO(OH) compared with the LiCoOO lattice. (*b*) shows the PDF calculated for model **1** using the LiCoOO (red) and CoO(OH) (green) lattices. The black line is the experimental PDF measured for the CoPi, normalized to the amplitudes of the calculated PDF from the amplitudes of the di-μ-oxo linked Co–Co peaks. The insets show an enlargement of the PDF around the di-μ-oxo linked Co–Co peak. The vertical lines highlight longer range Co—Co distance peaks in the experiment that are found to more closely match those in the LiCoOO domain compared with the CoO(OH) domain. The experimental peak at 10.08 Å is labelled by the Co1—Co11 and Co1—Co12 atom pair distances, annotated in (*a*), and correlates to the 10.15 Å peak for LiCoOO and the 10.28 Å peak for CoO(OH).

Finally, lattice layers for CoOOH and LiCoOO differ in the details of structure that are discernible as small differences in atom pair distances in the PDF. These can be compared with the PDF for the amorphous cobalt oxide OEC. Fig. 5 ▸(*a*) shows the overlap of the lattice structures for CoOOH and LiCoOO using the domain model **1**. CoOOH compared with LiCoOO has a slightly expanded domain size that occurs because of an increased di-μ-oxo linked Co—Co distance of 2.851 Å compared with 2.816 Å for LiCoOO. The PDF patterns for the two model domains are shown plotted in Fig. 5 ▸(*b*), along with the PDF measured for the CoPi. Remarkably, the PDF measured for the CoPi distinguishes between the CoOOH and LiCoOO lattice models. In particular, the LiCoOO model is found to provide a better match of the experimental di-μ-oxo linked Co—Co distance, and longer range Co—Co distances, including that between Co1 and Co11 (or Co12) annotated in Fig. 5 ▸(*a*), and positioned at 10.1 Å in the experimental PDF, compared with 10.15 Å for the LiCoOO PDF peak, and 10.26 Å for CoOOH. These distance differences are very small, but potentially of interest since they reflect structure changes arising from changes in electronic structure for the cobaltate layer. For this reason, the development of techniques that track the changes in amorphous oxide domain structures could provide insights into unravelling the sources for enhanced catalysis. Amorphous cobalt oxides are often described as cobalt oxyhydroxides (Farrow *et al.*, 2013 ▸; Gerken *et al.*, 2011 ▸), including from an analysis of X-ray emission spectroscopy (Friebel *et al.*, 2013 ▸). However, the PDF analysis here suggests that the oxyhydroxide and anionic cobaltate domains can be distinguished, and that the CoPi, CoMPi and CoBi OEC can be best described as anionic cobaltates with layer spacings in stacked assemblies, intermediate between those of the LiCoOO and CoOOH forms.

## 4. Conclusion   

**Figure 6:**
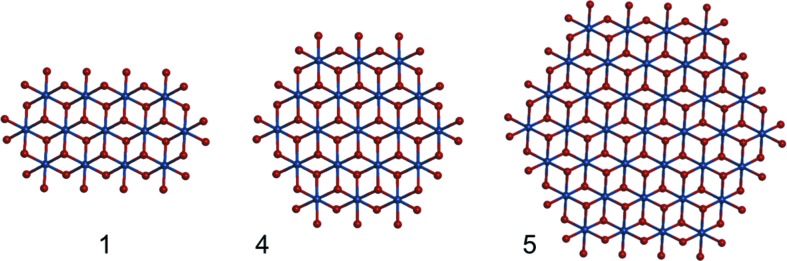
Scale of oxyanion-dependant changes in domain size for the amorphous cobalt oxide OEC. The LiCoOO form of model **1** was used for fitting the domain dimension for the CoPi. Models **4** and **5** provide the scale of domains corresponding to the CoMPi and CoBi OEC, respectively. In addition, as described in the text, the increase in the domain size for CoMPi and CoBi was correlated to increases in the extent of disordered layer stacking, with limited stacking coherence length, corresponding to mixtures of monolayers, dimers and trimers.

The X-ray scattering and PDF analysis has characterized the changes in domain structure for amorphous cobalt oxide OEC upon replacement of Pi with MPi and Bi. Previous PDF analysis of CoPi and CoBi OEC found that the replacement of the oxyanion was accompanied by an increase in domain size for the CoBi compared with the CoPi, and was correlated with the appearance of a disordered layered domain structure in the CoBi (Farrow *et al.*, 2013 ▸). In the present work, we used the high intensity, high X-ray energy beamline at APS to obtain PDF from scattering data with high signal-to-noise through the reciprocal space resolution up to 24 Å^−1^, and examined domain structures for cobalt OEC formed in the presence of the potassium salts of Pi, MPi and Bi. The films were found to be composed of analogous cobaltate domains that increase in size following the sequence of Pi < MPi < Bi, illustrated in Fig. 6 ▸. Further, we show that high-resolution PDF measurements are capable of distinguishing between cobalt oxyhydroxide and anionic cobaltate domains, and can resolve distortions in coordination geometries, modelled to be associated with the domain edge or defect sites. The results show that lattice structures in amorphous cobalt oxide OEC can be best correlated to those of the anionic cobaltates, distinguished from CoOOH. CoPi is found to reproducibly consist of monolayer domains, or with large, disordered layer spacing lying outside the measured scattering region. Coincident with the increase in domain size, CoMPi and CoBi are found to show progressively larger extents of stacking with layer spacing and inter-layer alignment corresponding to that in LiCoOO models. In addition, the series CoPi, CoMPi and CoBi shows a progressive decrease in contributions from distorted coordination geometries, consistent with the interpretation that these are associated with the domain edge. The results show that high-resolution scattering and PDF data can provide information on characteristics of amorphous oxide domain structures that are significant for correlation of amorphous oxide domain structures to catalytic activities.

## Supplementary Material

Supporting information is provided with 9 figures as described in the text.. DOI: 10.1107/S2052520615022180/ps5049sup1.pdf
