# Supplementary material for: Oxyanion induced variations in domain structure for amorphous cobalt oxide oxygen evolving catalysts, resolved by X-ray pair distribution function analysis
Source: Acta Crystallogr B Struct Sci Cryst Eng Mater. 2015 Dec 1;71(Pt 6):713–21. doi: 10.1107/S2052520615022180 (PMC4669998; doi:10.1107/S2052520615022180)
Supplement: Supplementary file 1 [file b-71-00713-sup1.pdf]

Supporting information

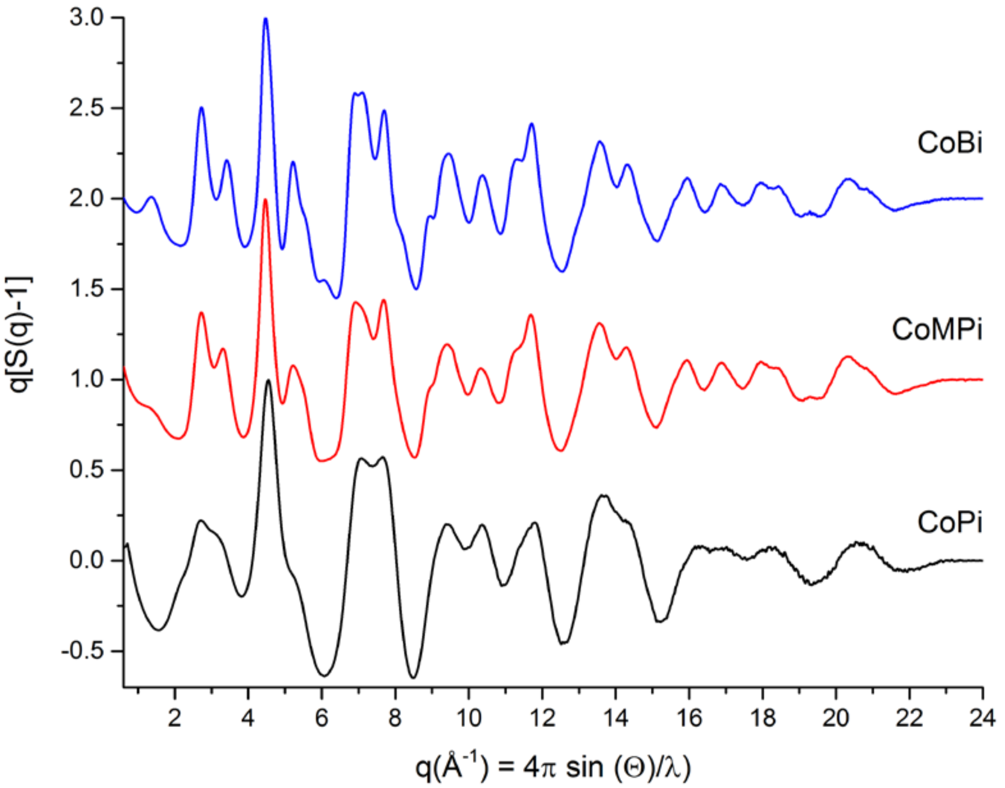

Figure S1 Experimental reduced scattering structure functions, F(q), measured for the cobalt oxide water oxidation catalysts *ex situ* films grown from three different buffer solutions described in the text, and used to calculate the PDF patterns shown in Figure 2. The F(q) traces are arbitrarily vertically offset to increase visibility, bottom, CoPi; middle, CoMPi; top, CoBi. The reduced scattering structure functions F(q) were calculated from the high energy X-ray scattering patterns, I(q), using equation **1** and as described in the text.

IMPORTANT: this document contains embedded data - to preserve data integrity, please ensure where possible that the IUCr Word tools (available from http://journals.iucr.org/services/docxtemplate/) are installed when editing this document.

A

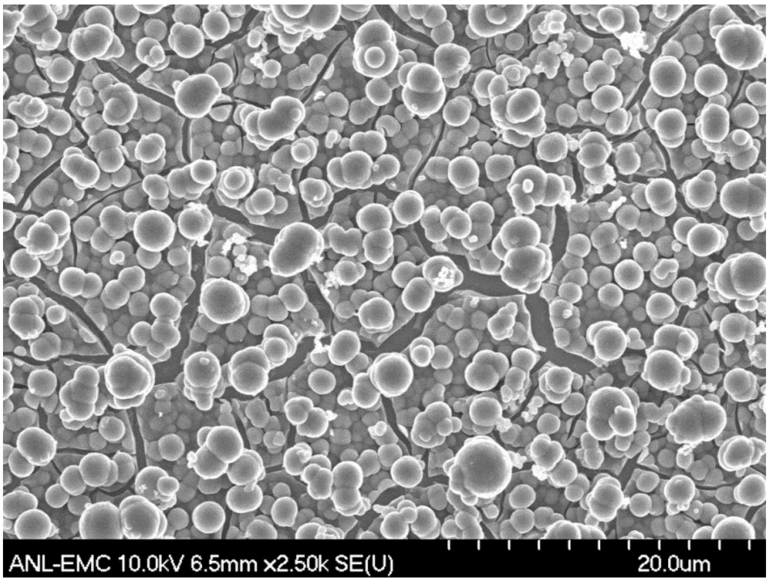

B

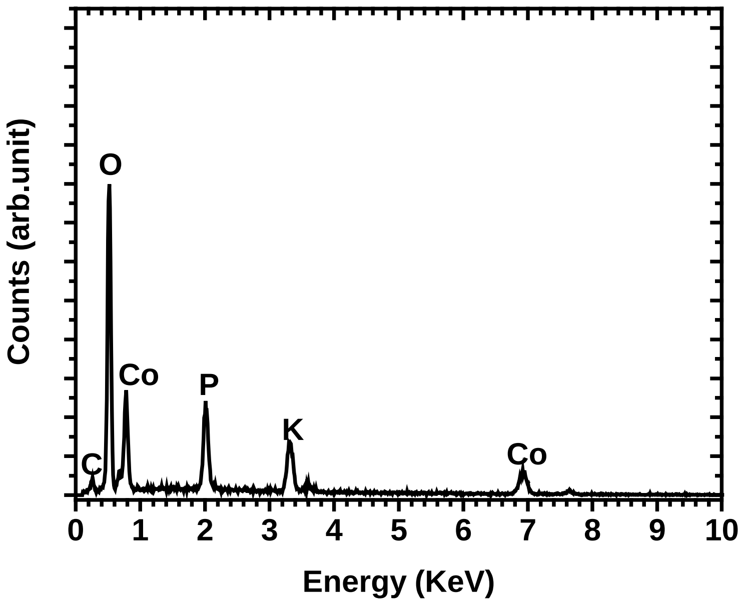

Figure S2 (A) Scanning electron micrograph, SEM, and (B) energy dispersive X-ray (EDX) spectrum measured for a CoPi-OEC, with corresponding PDF shown in figure S4. The Co:P:K ratio of the composition of the film is 2.8:1.2:1.

A

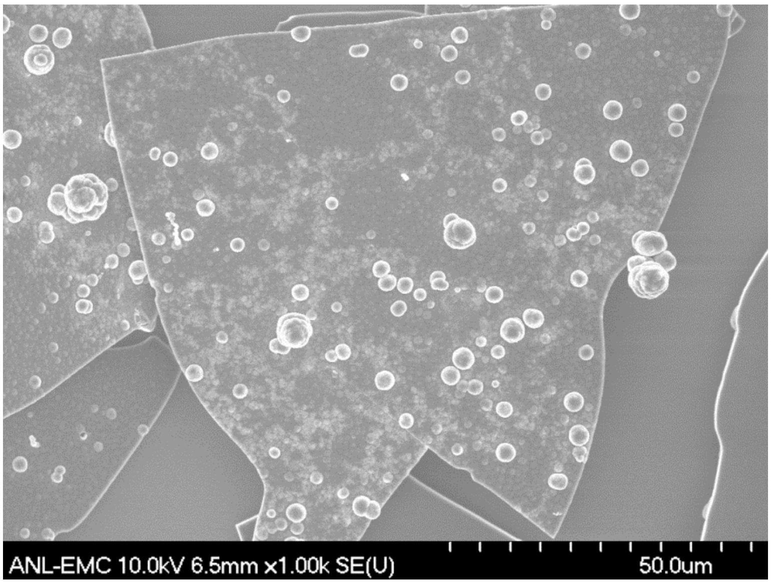

B

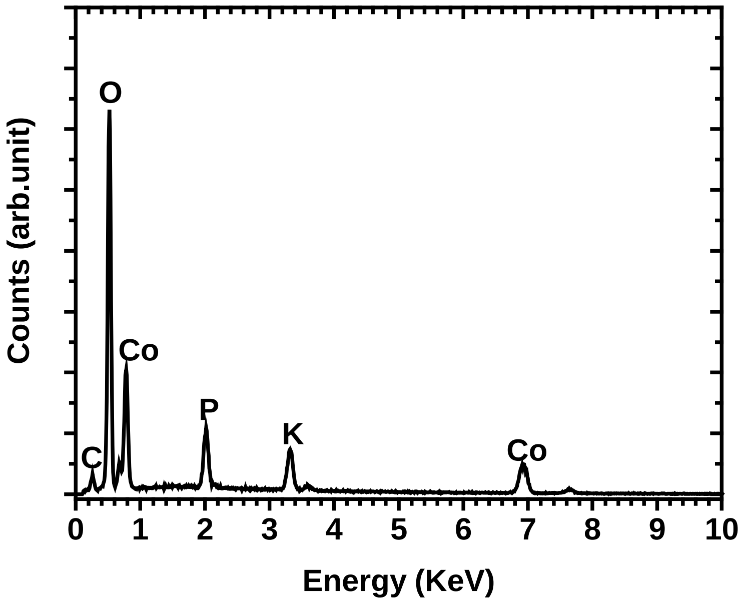

Figure S3 (A) Scanning electron micrograph, SEM, and (B) energy dispersive X-ray (EDX) spectrum measured for a CoMPi-OEC, with corresponding PDF shown in figure S4. The Co:P:K ratio of the composition of the film is 5.6:1.3:1.

A

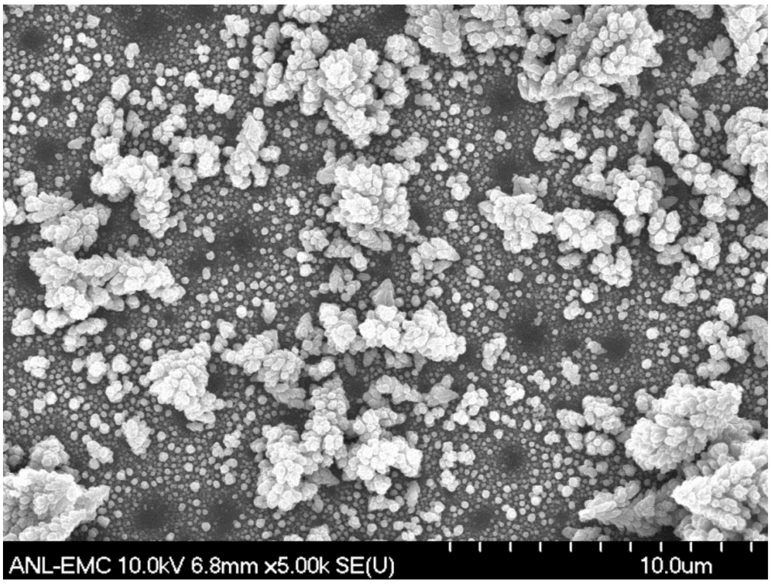

B

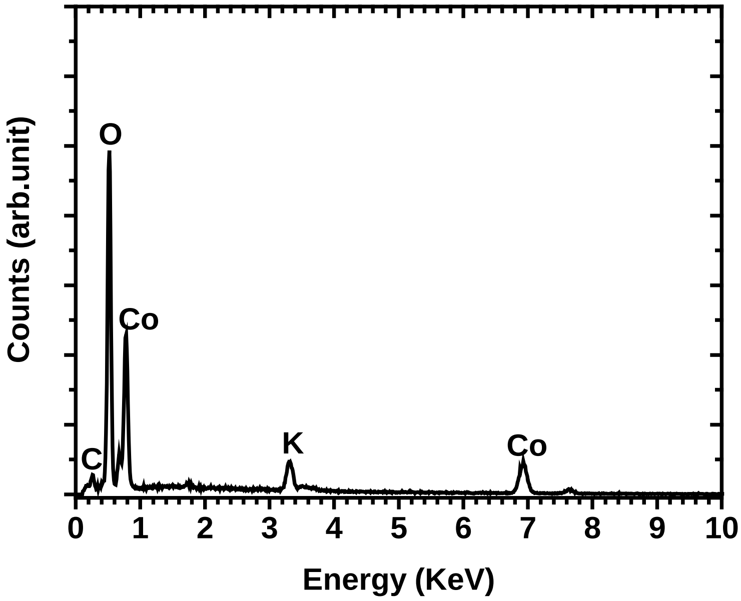

Figure S4 (A) Scanning electron micrograph, SEM, and (B) energy dispersive X-ray (EDX) spectrum measured for a CoBi-OEC, with corresponding PDF shown in figure S4. The Co:K ratio of the composition of the film is 10.9:1.

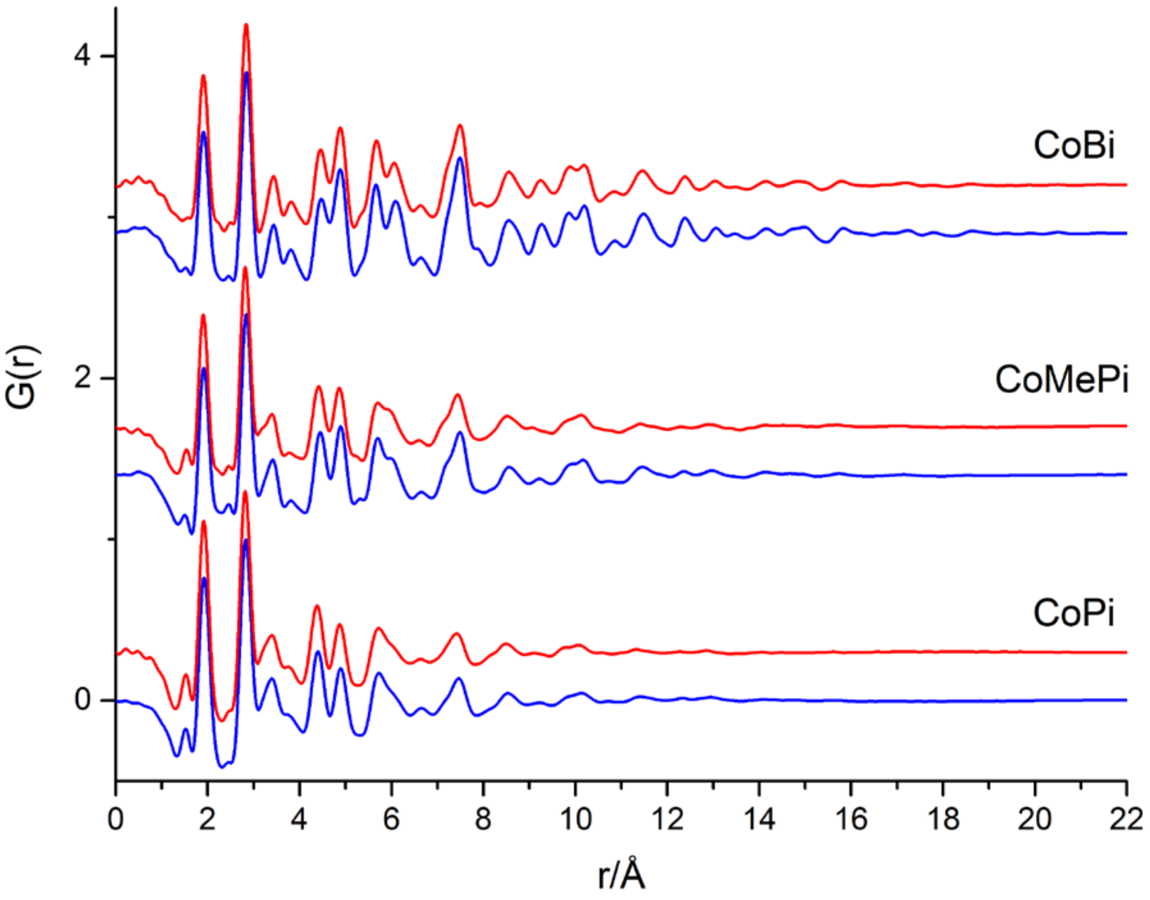

Figure S5 Examples of additional CoPi, CoMPi, and CoBi samples which show reproducibility in resolution of the P-O pair correlation peak in CoPi, as well as resolution of a small P-O peak amplitude in CoMP. The PDF patterns are normalized for the amplitude of the first shell CoO_6 peak at 1.9 Å and offset for clarity.

A

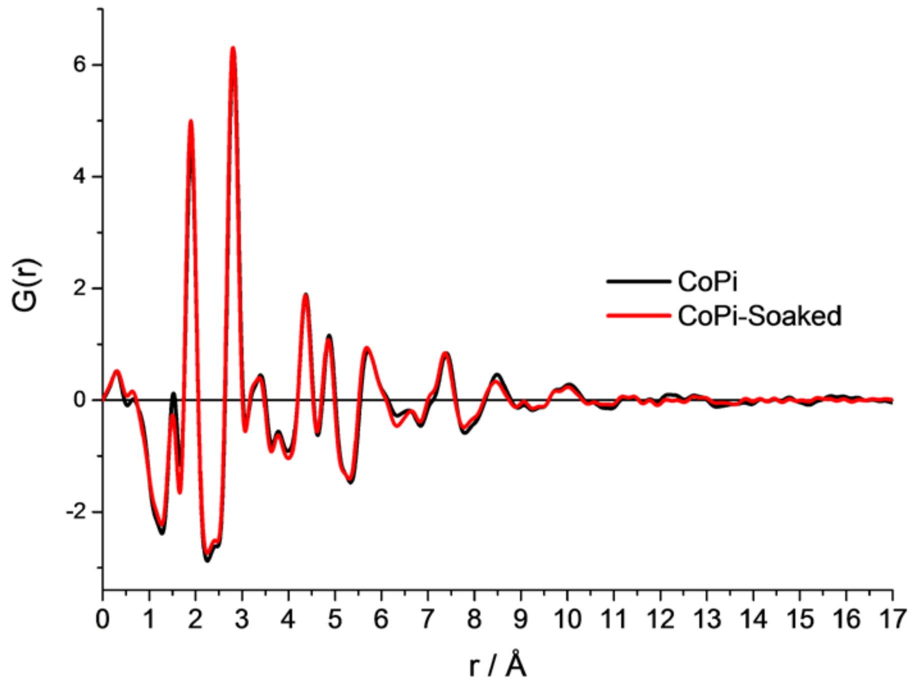

B

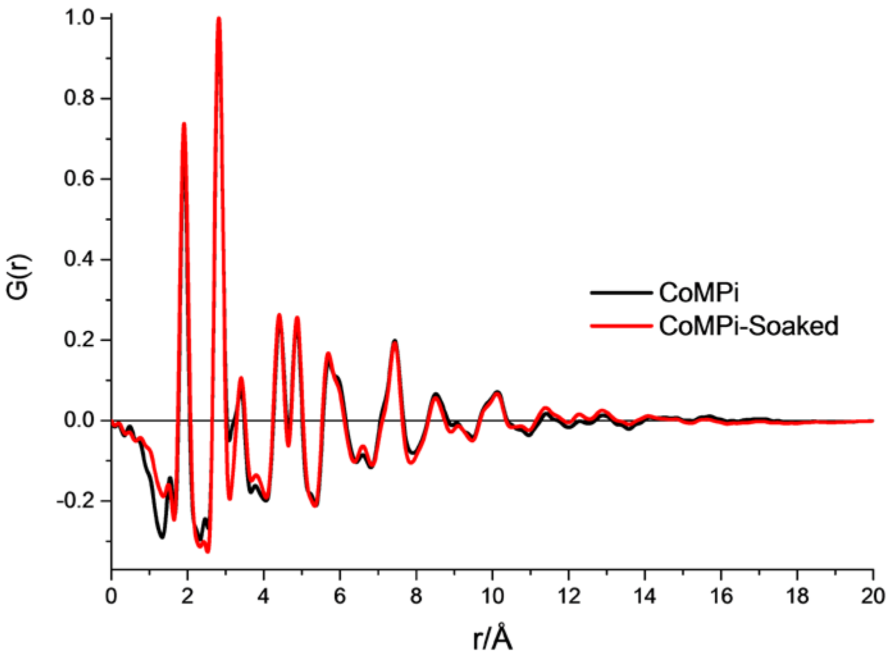

Figure S6 Comparison of PDF patterns for cobaltate OEC powders following either the standard procedure of simple rinsing or overnight soaking in Milli-Q purified water before removal of the electrodeposited films from electrode surfaces and X-ray measurements. Part A compares rinsed (black) versus soaked (red) CoPi. Part B compares rinsed (black) versus soaked (red) CoMPi.

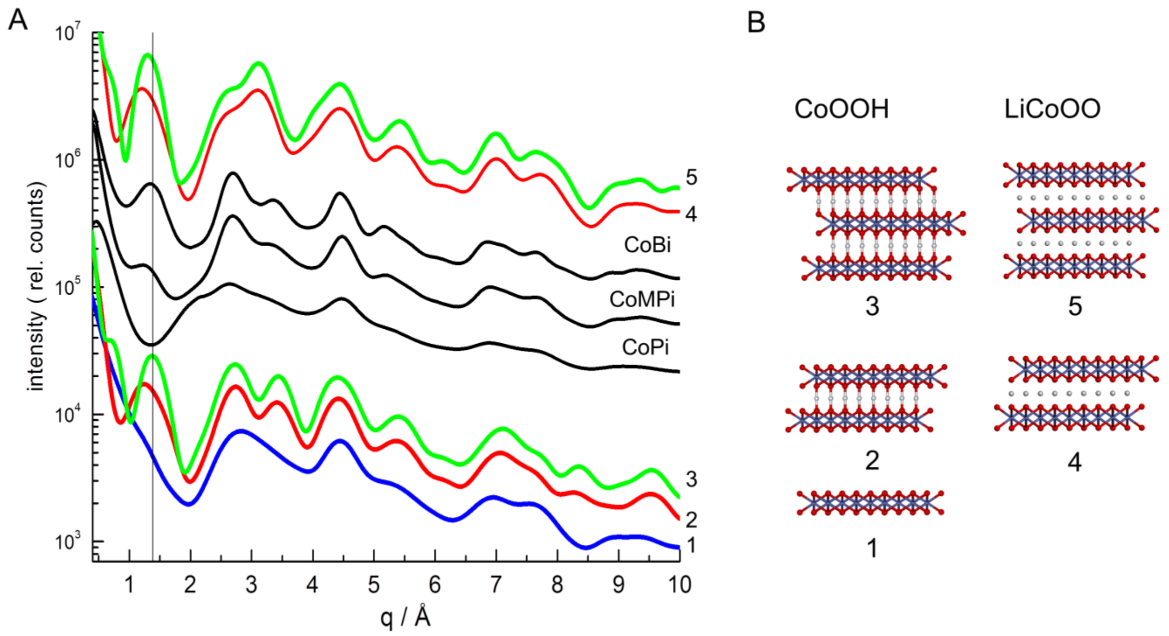

Figure S7 Part A. Comparison of experimental scattering I(q) for the CoPi, CoMPi, and CoBi OEC, middle traces, to scattering curves calculated from model domain structures. The curves labelled 1, 2, and 3 were calculated from 13 cobalt atom domains in a monolayer, bilayer, trilayer stack, respectively, and extracted from the CoO(OH) mineral structure. The top two curves, labelled 4 and 5, were calculated from 13 cobalt atom domains in a bilayer and trilayer stack, respectively, and extracted from the LiCoOO mineral structure. Side-on views of the model structures are show in Part B. For each mineral structure, the transition to a stacked structure is correlated with the appearance of a new interference peak in the scattering curve, with a position corresponding to the interlayer spacing and the number layers in the domain stacks.

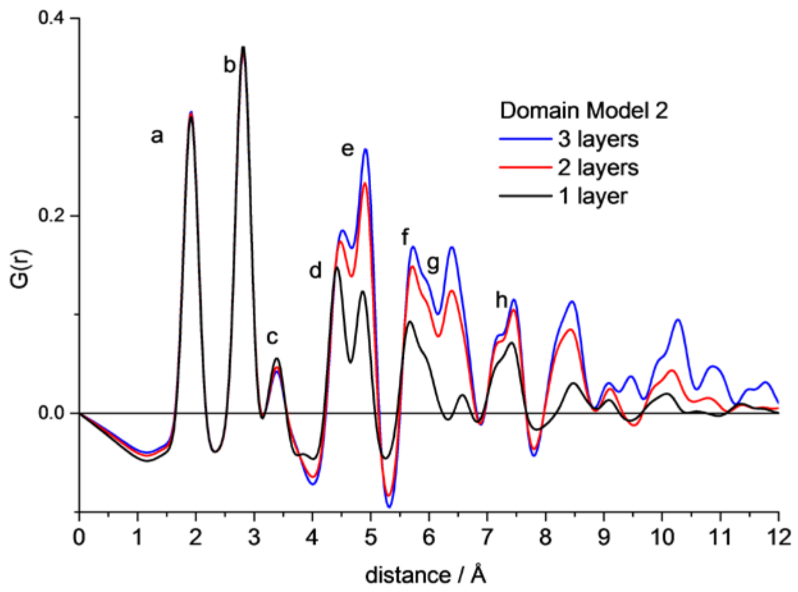

Figure S8 PDF calculated for monolayer (black), bilayer (red), and trilayer (blue) structures model **1** applied to the LiCoOO mineral structure, but with each layer having the distortions in coordination geometry for terminal oxygen atoms as shown in model **2.** The marker for stacking is still seen by the relative increase in the PDF peak labelled *e* compared to *d*. Further, the marker for edge atom distortions, identified by the loss of the amplitude for peaks *g* compared to *f*, is still detected in the PDF for the layered structures.

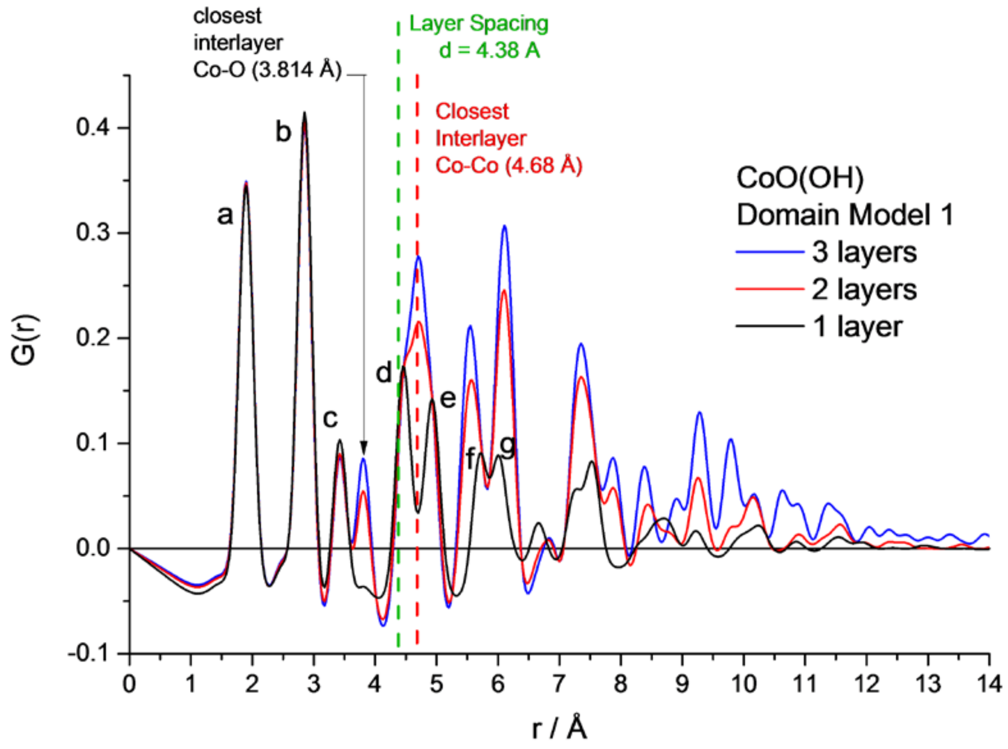

Figure S9 PDF calculated for monolayer (black), bilayer (red), and trilayer (blue) structures of model **1** applied to the CoOOH mineral structure. Characteristic of this mineral form, inter-layer pair distances in the layers structures are seen overlap and interfere with resolution of atom pair peak positions associated with the layer lattice structure. This is particularly noticeable for monolayer PDF peaks labelled *d* and *e*. Further, the inter-layer pair correlations also overlap the monolayer peaks labelled *f* and *g*, used to identify the presences of distortions to the coordination geometries for oxygen atoms at the domain edge. These PDF "markers" for stacking in the CoOOH are distinguished from those of LiCoOO, shown in Figure 4.
